# Bibliometric Insights in Genetic Factors of Substance-Related Disorders: Intellectual Developments, Turning Points, and Emerging Trends

**DOI:** 10.3389/fpsyt.2021.620489

**Published:** 2021-05-31

**Authors:** Kang Wang, Yijie Duan, Weicheng Duan, Yuxin Yu, Na Zheng, Jin Hu, Jia He, Haihong Chen, Man Liang

**Affiliations:** ^1^Department of Forensic Medicine, Tongji Medical College, Huazhong University of Science and Technology, Wuhan, China; ^2^Department of Endocrinology, Union Hospital, Tongji Medical College, Huazhong University of Science and Technology, Wuhan, China; ^3^Department of Pathology, Health Science Center, Shenzhen University, Shenzhen, China; ^4^Department of Otolaryngology-Head and Neck Surgery, Tongji Hospital, Tongji Medical College, Huazhong University of Science and Technology, Wuhan, China; ^5^Department of Public Health, Shihezi University School of Medicine, Shihezi, China; ^6^School of Health Policy and Management, Nanjing Medical University, Nanjing, China

**Keywords:** bibliometric, substance-related disorder, genetics, quantitative analysis, research frontiers

## Abstract

Substance-related disorders are a group of medical conditions that affect a person's brain and behavior and lead to an inability to control the use of legal or illegal drug(s) or medication. Substance-related disorder is a serious public health and society problem worldwide. Genetic factors have been proven to have an important role. Researchers have carried out a lot of work in this field, and a large number of research results have been published in academic journals around the world. However, there are few overviews of research progress, presentation, and development trends in this field. In this study, a total of 636 articles related to genetic factors of substance-related disorders were retrieved from the Web of Science (WoS) database from 1997 to 2018, and the scientific literatures were analyzed by bibliometrics. The study found that the United States (US) has maintained a leading position in the field of research, with many core institutions and plenty of high-quality research results. Alcohol use disorder is still the most concerning issue in this field. Over the past 20 years, new techniques such as genome-wide association study (GWAS) based on high-throughput sequencing technology have replaced family studies, twin studies, and retrospective studies in this field. We believe that it is urgent to study the genetic factors of substance-related disorders, which can greatly deepen the understanding of the pathogenesis of substance-related disorders and may provide potential targets for precise treatment of such diseases.

## Introduction

Substance abuse is a phenomenon where humans consume substances [either drugs (prescribed or free) or other substances], which are harmful to themselves or others, including both legal and illegal substances. The user's judgment, perception, attention, and body will often be out of control, and the dosage is usually much larger than the medical recommendation. Substance-related diseases, including substance dependence and substance abuse, are chronic relapsed diseases characterized by obsession and physical or psychological dependence (1, 2). These substances can induce a pleasurable “high” in addicts. Commonly abused substances include alcohol, heroin, cocaine, marijuana, and cigarettes, as well as other tobacco products, and some prescription (3) or over-the-counter (OTC) drugs, such as tramadol (4).

A worldwide survey performed in 2017 revealed that one in five adults was reported to have been drunk in the past month, and nearly one in seven adults had cigarette addiction (5). The report of the European Monitoring Centre for Drugs and Drug Addiction (EMCDDA) in 2018 shows that between 2012 and 2016, the number of reported drug-related deaths across the European Union (EU) is increasing, especially for people over the age of 30 (6). In addition, abuse and misuse of illegal drugs are recognized factors that contribute to the global burden of public health systems, and drug-injecting is still an important route of transmission for several diseases such as HIV or hepatitis C virus. The health burden caused by substance-related diseases induces and is accompanied by huge economic costs, including law enforcement, health care costs, and other direct and indirect costs, such as loss of productivity or harm to others (7).

Studies have shown that environmental and social factors, traumatic stress experience, and biological factors may influence the onset of substance-related disorders (8, 9). Among them, genetic factors have an important influence on the progression of substance-related disorders, especially alcohol, tobacco abuse, and nicotine dependence (10, 11). Research concentrated on family, twin, and adoption studies suggested that the familial heritability transmission of illicit substance use disorders is estimated to range from 30 to 80% (1, 12, 13). Genome-wide linkage studies and GWAS have demonstrated that alcohol dehydrogenase (ADH) and gamma-aminobutyric acid type A receptor subunit alpha2 (GABRA2) are associated with alcohol dependence (14, 15), while sigma-1 receptor (σ1R) is associated with methamphetamine (METH) abuse (16). Besides, several chromosomal loci related to drug abuse vulnerability were identified through reanalyses of genome scanning data of illegal drug addiction (17).

Due to serious social harmfulness and the accompanying social burden, research on substance-related disorders has been a continuous concern. The bibliometrics method facilitates to analyze quantitatively collections of scientific literature and visible aspects of science dynamics in sets of scholarly communication. By analyzing the influence of journals, institutions, highly cited references, and high-frequency terms, classifying and clustering them from a technical level, it can provide an overview of the research status and development trend of this field and help researchers to grasp the key points of future development (18). In this way, we will deepen our understanding for genetic factors of substance-related disorders and promote more benign developments of research.

## Data Source and Methodology

### Database Building and Searching Strategy

As one of the most famous academic databases, the Web of Science (WoS) Core Collection database was used, which gathers a collection of more than 20,000 journal headlines and fully indexed cited references, authors, and author affiliations. Thus, an initial database has been built in which all papers retrieved with a specific query were stored and further handled. Medical Subject Headings (MeSH) list was established by the National Library of Medicine and is used for indexing, cataloging, and searching of biomedical and health-related information. The entry searching terms of “Substance-Related Disorders” and “Genetics” came from a standardized MeSH list (Supplementary Table 1) to make a complete retrieval coverage in the topic method and minimize both false-positive and false-negative results for bibliometric analysis as possible. This search strategy identified 931 records over all the previous years up to August 6, 2019.

### Post-processing of Scientific Literature Acquired and Bibliometric Analysis Mapping

**Figure 1:**
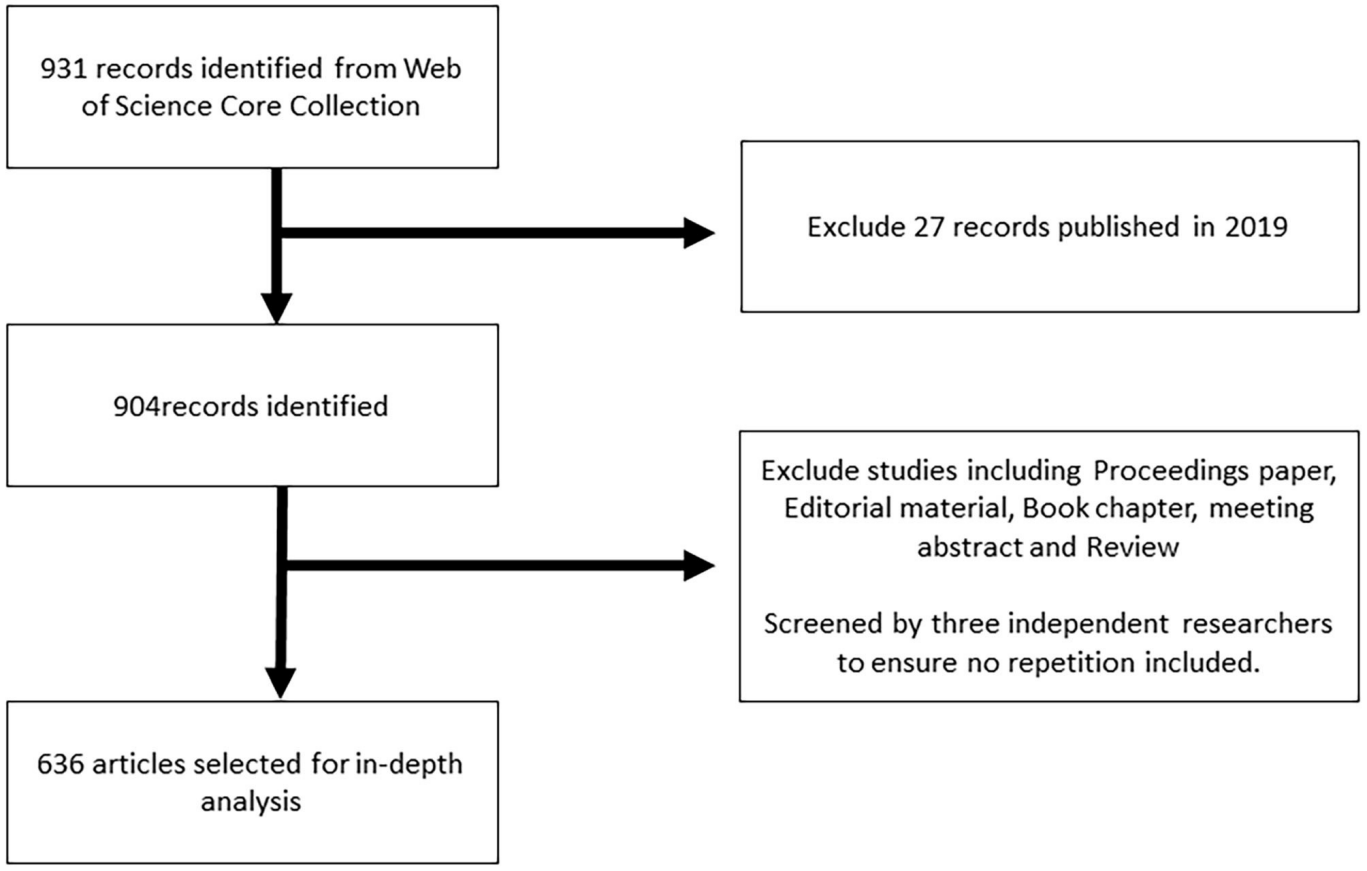
The flowchart of the methodology.

Eventually, proceedings paper, note, editorial material, book chapter, letter, reviews, meeting abstract, and correction were excluded from the database. Only articles published in scientific journals were included in this analysis without restrictions based on language or subject area, which finally reduced the set to 636 records. VOSviewer 1.6.9 and CiteSpace 5.5 are tools which allow the analysis and visualization of bibliometric networks in order to detect structures and changes in the research field. As we attempted to retrieve all the literature from 1986 to 2018, the results of distribution by years indicate that the literature in this field was first published in 1997. A total of 931 records were finally retrieved from the WoS Core Collection database, and the records retrieved were screened by three independent researchers with different major backgrounds. Only original articles were downloaded for other document types either equipped with inflated citing scores such as reviews or created little new worthwhile knowledge such as comments (Figure 1). All documents were retrieved and downloaded within the same day to avoid any changes caused by daily database updates.

We identify the retrieved scientific literature distribution on time-publication outputs, notice and capture the collaborations between countries/territories, and develop an analysis of keywords endured citation burst and shift from early research field to the recent hot topics. The countries/territories were assigned according to the WoS Core Collection database, incorporating all listed countries in the publication without any consideration of the order of authors. Co-citation study is defined as a relationship of co-citation when two articles were cited by the third document, which is regarded as a commonly used method to quantitatively visualize maps.

## Results

### Development of Publications

**Figure 2:**
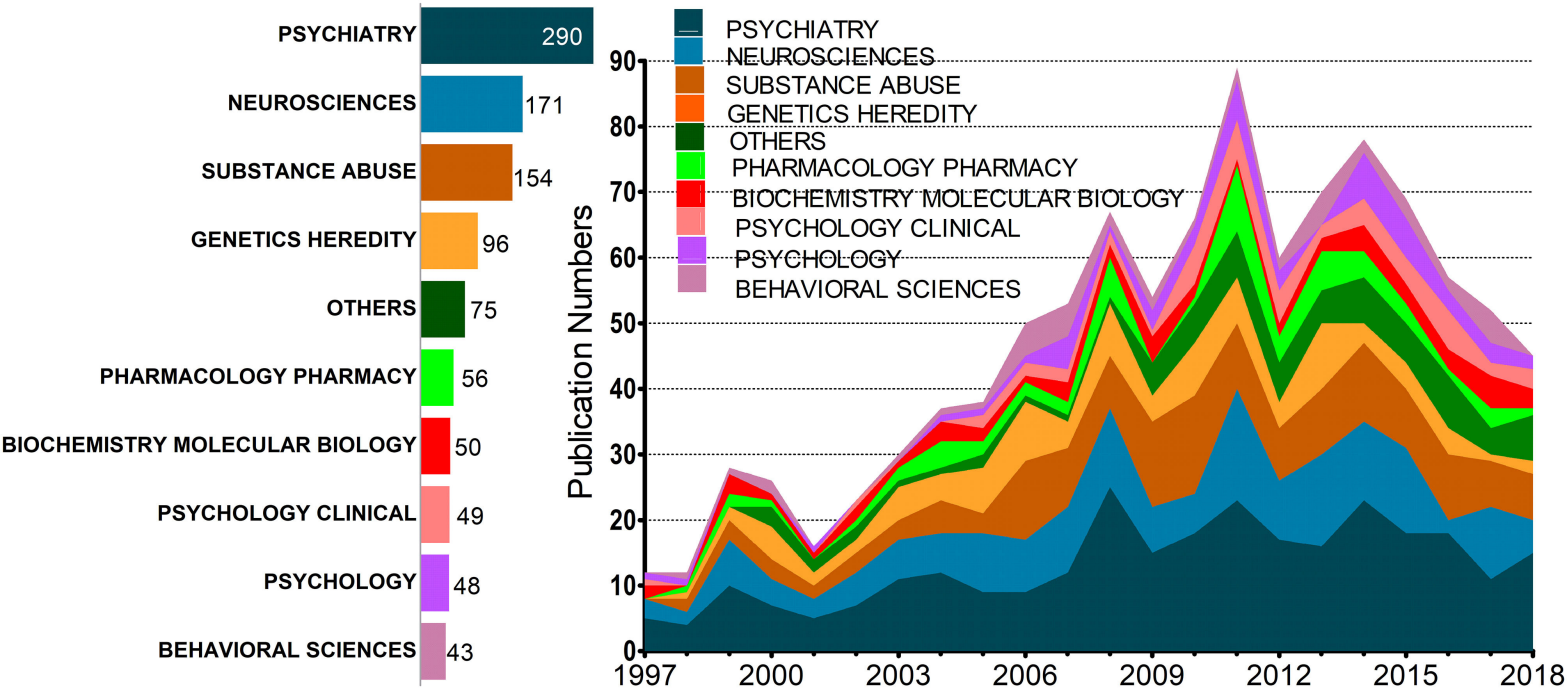
Distribution of the annual number of substance-related disorders publications from 1997 to 2018; publications of different subjects are characterized by colors.

Annual publications per year per publication from 1997 to 2018 are summarized (Figure 2). In these 22 years, the growth rate fluctuates with the annual number of publications increased over time and the publication number increased nearly seven-fold from 12 records in 1997 to 89 in 2011, the year when peak publications were observed.

### Leading Countries or Regions

**Figure 3:**
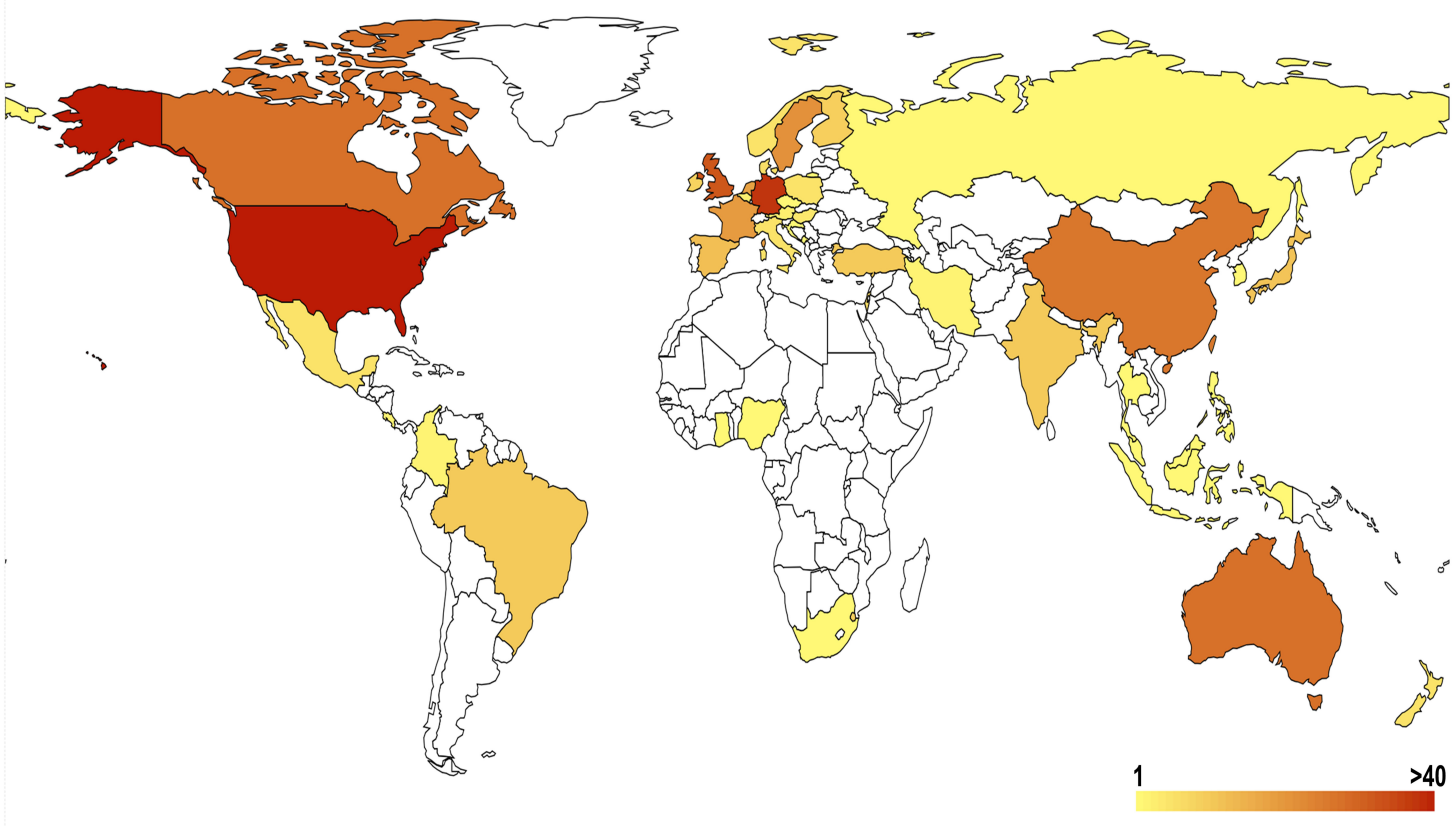
Geographical distribution of substance-related disorders publications. Redness degree is positively correlated with publication numbers.

**Figure 4:**
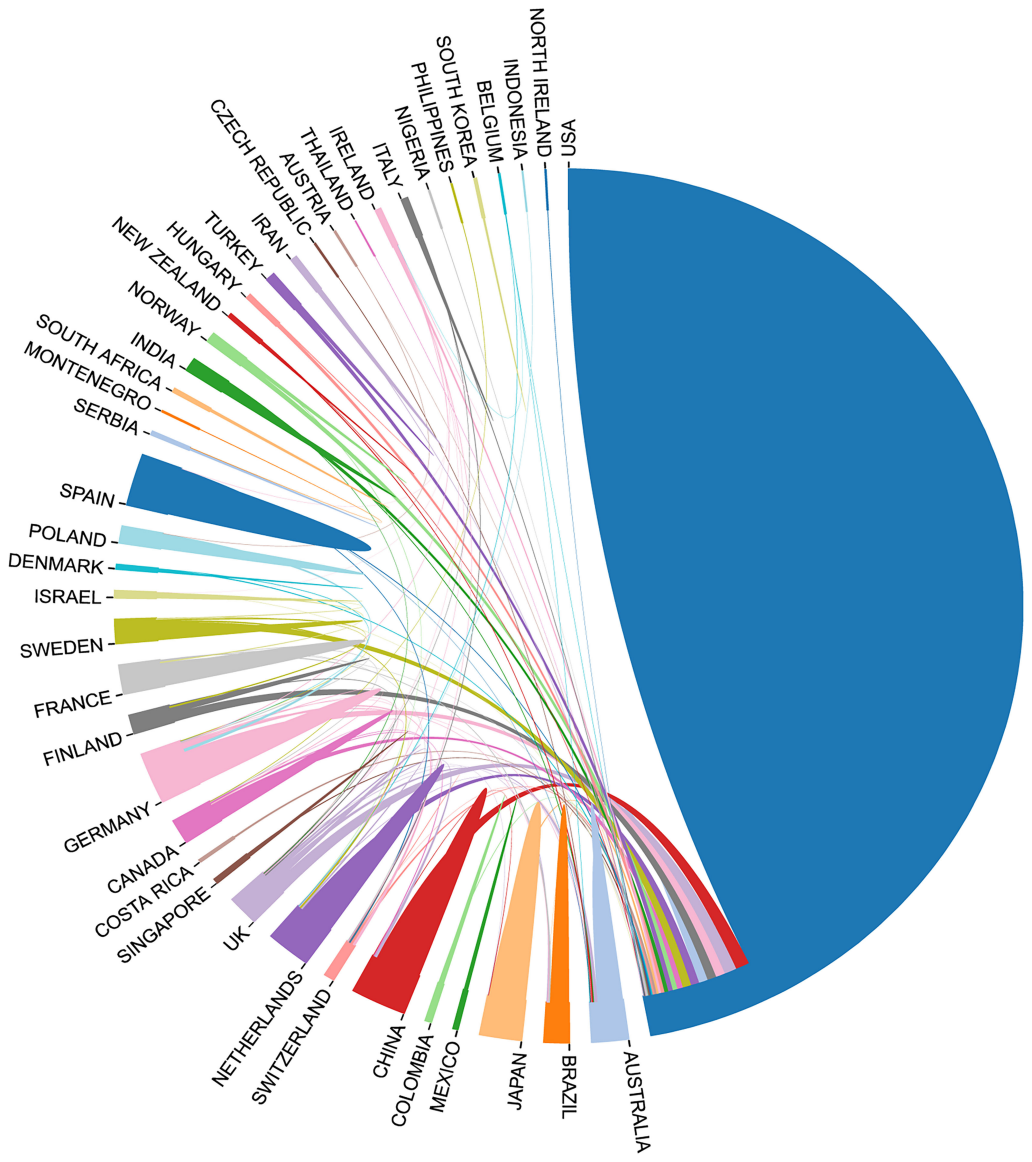
Cooperation between contributed countries.

The number of publications from a country or region is an important indicator that reflects the attention placed on the field and the research strength in the specific research area. At least 41 countries or regions contributed to scientific research on genetic factors of substance-related disorders (Figures 3, 4). Fourteen countries contributed >10 articles and seven countries contributed >20 articles. The US has contributed the most publications and has extensive cooperation with many other countries, being undoubtedly dominant in the research field. Also, it had the highest h-index of 70, followed by Germany (19) and England (17). The highest citation/article ratio was reached with 49.84 in Germany, 37.49 in the US, and 36.56 in the Netherlands. The most productive institutions were the University of California System (93; 18.90% of US documents), which had contributed more publications than any other institutions (Table 1).

**Table 1:** Top countries and country institutions on substance-related disorders research.

**Country**	**Articles**	**Citations**	**H-index**	**Citations per article**	**Top country institution**	**Top institution articles (%)**
USA	492	18,445	70	37.49	University of California System	93 (18.90%)
Germany	37	1,844	22	49.84	Central Institute of Mental Health	10 (27.02%)
England	32	1,152	16	36.00	University of London	23 (71.87%)
Australia	27	496	14	18.37	Washington University WUSTL	10 (37.03%)
Canada	27	855	11	31.67	University of Toronto	10 (37.03%)
China	26	636	13	24.46	Peking University	7 (26.92%)
Sweden	21	457	10	21.76	Karolinska Institutet	11 (52.38%)
France	19	358	11	18.84	Institut National de la Sante et de la Recherche Medicale Inserm	12 (63.15%)
Netherlands	18	658	10	36.56	Radboud University Nijmegen	10 (55.55%)
Spain	12	275	7	22.92	Ciber Centro de Investigacion Biomedica en Red	7 (58.33%)

### Most Active Journals

More than 220 scholarly journals have published articles on genetic factors of substance-related disorders research. The top 10 most active journals have contributed 33.61% records in sum and exactly meet the “core” journal standard according to Bradford's law (20). For the whole retrieved database, the “core” journals included the following: *Alcoholism Clinical and Experimental Research, Drug and Alcohol Dependence, American Journal of Medical Genetics Part B-Neuropsychiatric Genetics, Molecular Psychiatry, Addiction, Addiction Biology, Psychological Medicine, Psychiatric Genetics, Behavior Genetics*, and *Biological Psychiatry* (Table 2). The journals are indexed in Journal Citation Reports (JCR) as well as their impact factor 2018, JCR quartile, and WoS categories. Among these journals, six journals are classified into the psychiatry category, four into the substance abuse category, and three into neurosciences and genetics and heredity categories.

**Table 2:** The 10 most active journals that published articles on substance-related disorders research.

**Journal**	**Published numbers (%)**	**IF** **2018**	**SJR** **2018**	**JCR** **quartile**	**Categories**
*Alcohol Clin Exp Res*	32 (5.03%)	3.235	1.46	Q2	Substance abuse
*Drug Alcohol Depen*	32 (5.03%)	3.466	1.82	Q1	Psychiatry; substance abuse
*Am J Med Genet B*	28 (4.40%)	3.123	1.56	Q2	Genetics and heredity; psychiatry
*Mol Psychiatr*	21 (3.30%)	11.973	5.99	Q1	Biochemistry and molecular biology; neurosciences; psychiatry
*Addiction*	20 (3.14%)	6.851	2.78	Q1	Psychiatry; substance abuse
*Addict Biol*	19 (2.98%)	4.223	1.76	Q1	Substance abuse; biochemistry and molecular biology
*Psychol Med*	18 (2.83%)	5.641	3.08	Q1	Psychiatry; psychology; psychology, clinical
*Psychiat Genet*	16 (2.50%)	1.375	0.55	Q4	Genetics and heredity; neurosciences
*Behav Genet*	14 (2.20%)	2.313	1.11	Q2	Behavioral sciences; psychology, multidisciplinary; genetics and heredity
*Biol Psychiat*	14 (2.20%)	11.501	5.76	Q1	Neurosciences; psychiatry

### The Most Cited Publications in Substance-Related Disorders

Among the retrieved 636 science literature, 605 items were cited and 31 items were non-cited until August 6, 2019. It had 21,889 citations all together and an average of 34.42 citations per item with *h*-index = 72. The top 10 most cited publications in substance-related disorders are listed including titles, countries, published year, number of citations, and JCR impact factor 2018 (Table 3). All of these publications were published before 2010 and two publications were published before 2000. Kreek, MJ (Nat Neurosci, 2005) is the top-cited reference (585 citations), followed by Hesselbrock, M (Addiction, 1999) and Oslin, DW (Neuropsychopharmacol, 2003) (21–23). Nine out of the top 10 articles came from American authors and only one record came from Germany. Seven out of the 10 most frequently cited articles were published in journals with an impact factor >6.

**Table 3:** The characteristics of the highly cited and the most impactful classic articles.

**Rank**	**Total citations**	**Journal**	**Published year**	**Country**	**IF 2018**	**Article title**
1	585	*Nat Neurosci*	2005	USA	21.126	Genetic influences on impulsivity, risk taking, stress responsivity and vulnerability to drug abuse and addiction
2	532	*Addiction*	1999	USA	6.851	A validity study of the SSAGA—a comparison with the SCAN
3	420	*Neuropsychopharmacol*	2003	USA	7.16	A functional polymorphism of the mu-opioid receptor gene is associated with naltrexone response in alcohol-dependent patients
4	388	*Pharmacol Biochem Behav*	2000	USA	2.773	Drinks like a fish: zebra fish (Danio rerio) as a behavior genetic model to study alcohol effects
5	310	*P Natl Acad Sci USA*	2010	USA	9.58	A genome-wide association study of alcohol dependence
6	307	*Arch Gen Psychiat*	1998	USA	14.48	Familial transmission of substance dependence: alcohol, marijuana, cocaine, and habitual smoking—a report from the collaborative study on the genetics of alcoholism
7	293	*Alcohol Res Health*	2007	USA	1.577	The genetics of alcohol metabolism—role of alcohol dehydrogenase and aldehyde dehydrogenase variants
8	239	*J Neural Transm*	2008	Germany	2.903	Molecular genetics of adult ADHD: converging evidence from genome-wide association and extended pedigree linkage studies
9	234	*Biol Psychiat*	2002	USA	11.501	The borderline diagnosis II: biology, genetics, and clinical course
10	230	*Mol Psychiatr*	2009	USA	11.973	Genetic variation in components of dopamine neurotransmission impacts ventral striatal reactivity associated with impulsivity

### The Landscape of Research Branches

**Figure 5:**
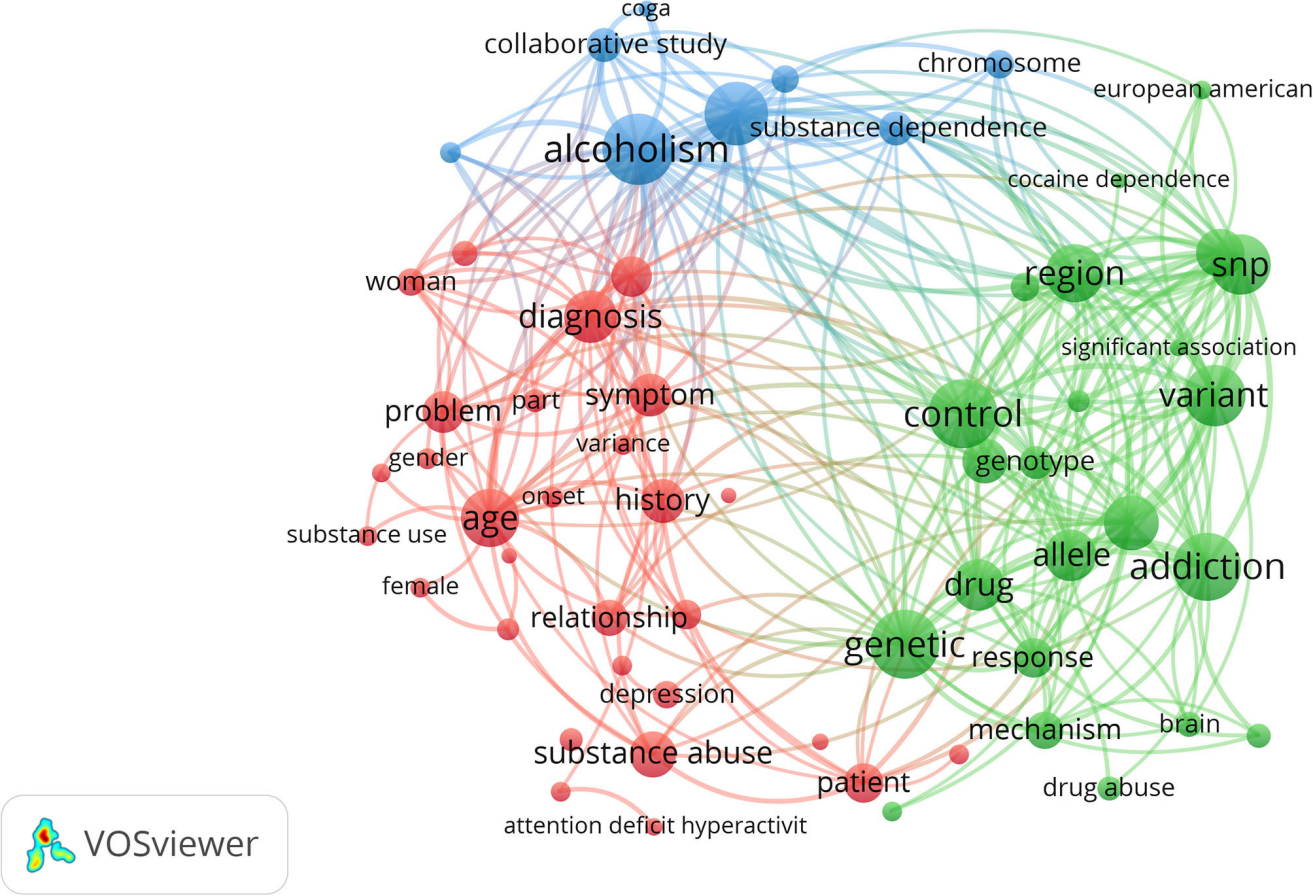
Co-occurrence network of terms of substance-related disorders publications, from 1997 to 2018. Terms are automatically extracted from titles and abstracts and divided into three clusters by natural language processing techniques of VOSviewer.

After removing general noun phrases, constructing and visualizing a co-occurrence network of terms extracted from the title and abstract of publication will help outline the central issue of this branch of research (19). Since terms that co-occur closely tend to be located close to each other during the visualization process, three main research areas were screened out and presented in red, green, and blue, respectively. “Control,” “Alcoholism,” and “Diagnosis” occupied notable positions of the graph with strong linkage with other terms (Figure 5). Other terms such as “Genetic,” “single nucleotide polymorphism (SNP),” and “Age” also play important roles in the research areas.

### Co-cited Reference Analysis

**Figure 6:**
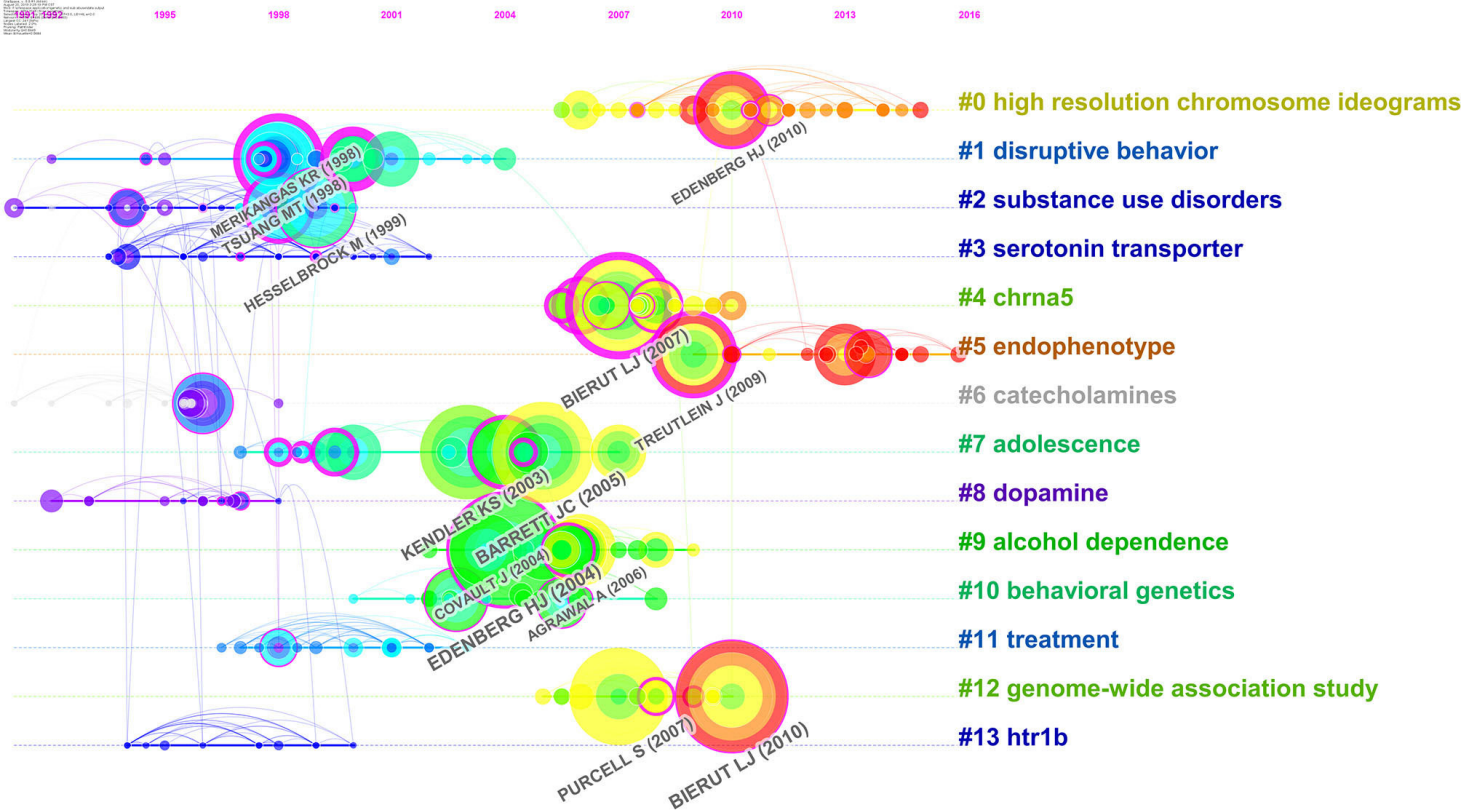
Co-cited reference timeline map based on CiteSpace. Co-cited reference that is commonly cited in the substance-related disorders literature are clustered and identified by CiteSpace. Years are arranged horizontally at the top, and the label of each cluster is shown at the end of the cluster's timeline.

To draw the development path of genetic factors of substance-related disorders, a co-cited reference timeline map was visualized (Figure 6). It presents both the publication years of the scientific literature and the clusters the terms belong to. Large-sized nodes are particularly worthy of attention because they are highly cited, especially nodes with red tree-rings presenting citation bursts (24). *High-resolution chromosome ideograms, endophenotype*, and *genome-wide association study* are the three clusters with higher citations recently. *Cholinergic receptor nicotinic alpha 5 subunit (CHRNA5), adolescence, alcohol dependence*, and *behavioral genetics* are the clusters in the middle of the timelines and other clusters appear in the earlier years.

### Research Frontiers Detection

**Figure 7:**
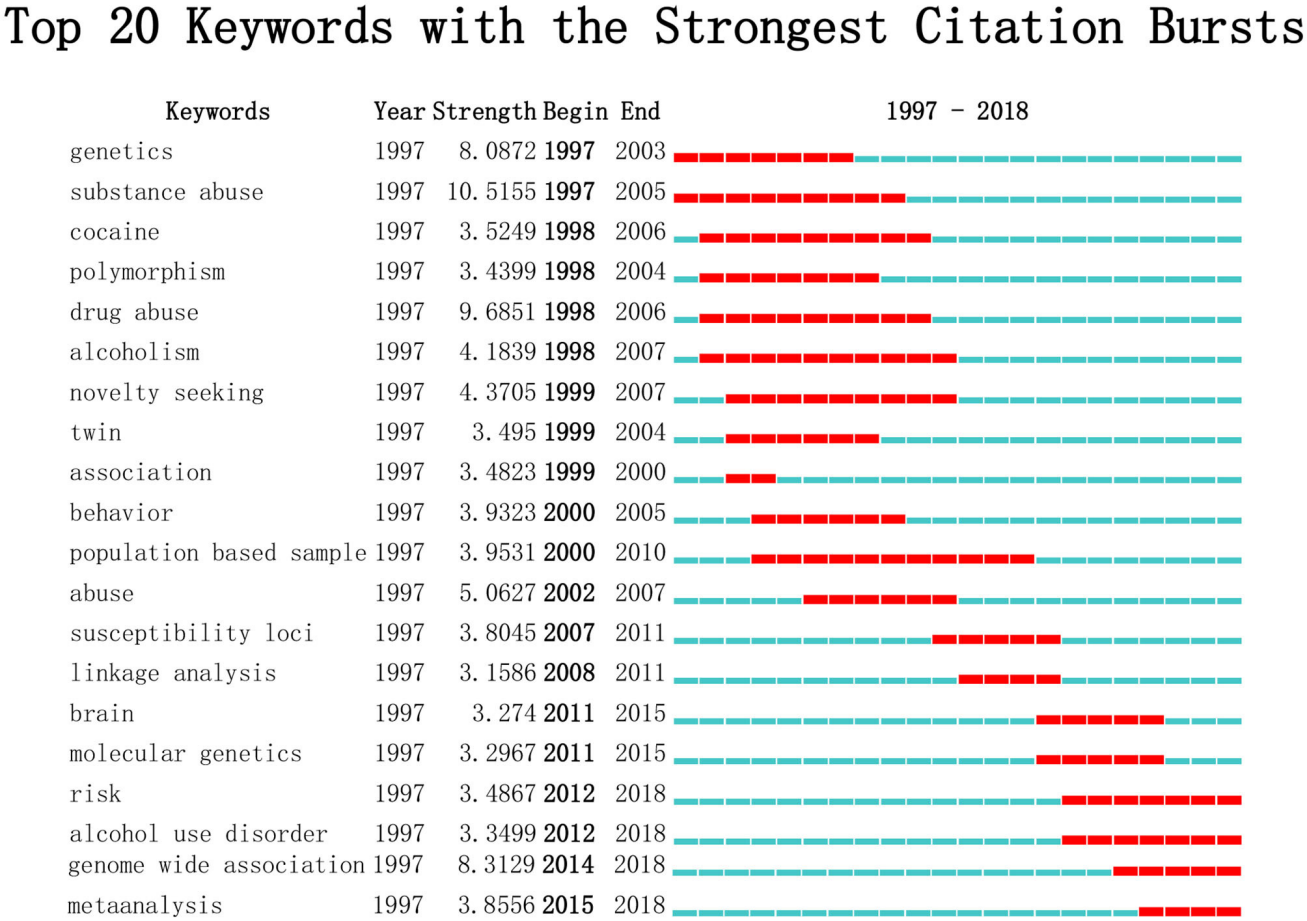
Top 20 keywords with the strongest citation bursts. Each short line represents a year and the line in red stands for the burst detection years. Keywords with red lines extending to the latest year can indicate the research frontiers in a short period of time in the future.

“Burst words” refer to words with significant growth characteristics in a certain field over a period, which are regarded as indicators of cutting-edge research topics. By detecting burst words through CiteSpace, major changes in research direction can be revealed (25, 26) (Figure 7). Since 2011, “brain,” “molecular genetics,” “risk,” “alcohol use disorder,” “genome-wide association,” and “meta-analysis” successively ranked on the burst list and the last four keywords kept bursting to the present.

## Discussion

By analyzing articles, books, and other publications with statistical methods, bibliometric methods are frequently used in the field of information science. Additionally, these methods can be applied to explore and evaluate researcher contributions across countries (27), their impact on the field, and particular impactful papers and to identify the research focus and predict frontiers in the coming years within a specific field.

Characterized by compulsive drug seeking, relapse, and persistent use despite serious and harmful consequences (28), substance-related disorder, as a common chronic brain disease, will bring impulsivity and aggression. It accounts for a loss of 13 disability-adjusted life years (DALYs) per 1,000 population worldwide, approximately 11 DALYs per 1,000 population are lost due to alcohol use, and approximately 2 DALYs are lost due to illicit drug use (29). Behavior studies proved that phenotypic variation in substance-related disorders is determined by genetics as well as different environmental conditions (30). In this study, we have performed a bibliometric analysis to explore worldwide trends in genetic factors of substance-related disorders research across 22 years, from 1997 when the first science literature was retrieved until 2018. To the best of our knowledge, this study is the first to offer insights into the research topics and trend evaluation in terms of genetic factors of substance-related disorders.

According to our search strategy, 636 academic papers conforming to the standard were retrieved from 1997 to 2018, with an average of 29 articles per year. Through analysis of the annual quantity of papers published, we found that the number of research papers in this field has been increasing year by year since 1997 and peaked in 2011. The results reflect that the problem has gradually been taken seriously, and more and more researchers and funds have been devoted in relevant research. All of these publications are contributed by institutions from 48 countries, and researchers from the US contributed nearly half of them. Besides, the papers from Germany are also very eye-catching because of the highest citation rate (Table 1). Through the analysis of the authors' nationality, we find that these studies are often carried out through international cooperation (Figure 4). Among them, the participation of the US is still the highest. In summary, substance-related disorders are a global issue, and the US contributes the most in related research fields.

As one of the three most important bibliometric laws, Bradford's law is used to measure the scattering of scientific information (31). The model proposed by Bradford consists of concentric zones (Bradford's zones) arranged in decreasing order of productivity and can be used to identify the core journals in a research field (32). The journals listed have published almost one-third of scientific literature in the field: six journals located in JCR quartile one, three in JCR quartile two, and only one in JCR quartile four (Table 2). This may illustrate that most of the core journals are considered high-quality grades through the JCR evaluation system. Due to major advances in genetics and neuroscience research, the understanding of the pathogenesis of substance-related disorders has been significantly deepened in the last 30 years (33). Neurotransmitter systems encoded by related genes are interactively involved in the acute or chronic effects of the most abused drug and constitute the basis of addiction and the initiating factors of substance abuse (23). On the other hand, exposure to abused substances can cause irreversible changes in neurons, thereby affecting the behavior (23).

Among the top 10 cited publications obtained by bibliometric analysis (Table 3), six publications have been cited more than 300 times, five were published in journals with an impact factor around 10, and the US contributed 9 out of 10. This indicated that the research field related to substance-related disorders continues to attract the attention of researchers, and researchers from the US are the main force in this field. In terms of the substance abuse involved, six of them focused on alcohol dependence, and the other four focused on other drugs or mechanisms. This shows that alcohol dependence remains the most concerning part of the field compared with other drug addictions, which is consistent with the severity of alcohol abuse and dependence problems worldwide. As a serious type of substance-related disorder, alcohol abuse involves a series of unhealthy drinking behaviors (34). Besides, it is the seventh leading risk factor in terms of disability-adjusted life years globally and accounts for a higher burden of disease than any other drug except for tobacco (35, 36).

Using natural language processing techniques, the VOSviewer extracts terms from the corpus file, where a term is defined as a sequence of nouns or noun phrases that can be found in a sentence. Analyzing the terms used by the authors and the database used for indexing purposes can help identify the core topics of a particular research area. Over the past 22 years, SNP, variant, chromosome, age, and gender have become active research topics related to substance-related disorders. The terms were classified into three prominent modules automatically by VOSviewer, and we summarized the topics as follows: genetic mechanism, epidemiology or sociology, and study of alcoholism, which represent the main research branches on genetic factors of substance-related disorder. Alcohol dependence is the leading component of a substance-related disorder, and GWAS and post-GWAS analyses have deepened our understanding of the SNP-related genetic etiology of alcohol dependence (37). Gender differences also affect the extent of substance abuse. Because of higher social pressure, men have a higher probability to develop substance addiction than women (38). Substance-related disorder is not only a scientific problem but a serious social problem as well, and it remains one of the major challenges to human society.

The research topic clustering technology is the prominent feature of bibliometrics. It categorizes all cited references in retrieved literature and automatically categorizes documents with similar topics (39). The timespan foundation of a research subtopic can be easily revealed by the timeline visualization map (Figure 6). The evolutionary process of genetic factors of substance-related disorders research can be identified and clues of future research directions in this field could be grasped. Through the clustering and visualization of the key literature in the field of substance-related barriers, it can be found that the research in this field can be divided into three distinct stages of development. Prior to 2000, researchers found that individuals with a family history had a significantly increased risk of substance-related disorders through family studies, twin studies, and retrospective studies. Therefore, family history is considered to be the most important potential risk in the development of such diseases (21, 40, 41). The literature between 2000 and 2007 showed that more attention was paid on *GABRA2* and *CHRNA5*. The risk of *GABRA2* mutations in adolescents produced externalizing problems to the development of problematic alcohol and drug use by multiple pathways (42, 43). Variants in *GABRA2* are associated with alcohol and nicotine dependence, representing an early risk factor to the problematic alcohol or drug use (42, 44–46). *CHRNA5* was considered to be highly associated with nicotine addiction (47), given the strong link between smoking and drinking, which pointed to a potential mechanism of alcohol dependence. Since 2007, GWAS began to be used to detect SNPs associated with substance-related diseases (48–50) and gradually became a common method and led the direction of research hot spots in this field (12, 51–53). With more high-resolution chromosome ideograms of substance-related disorders provided by GWAS, this facilitates investigators, physicians, and geneticists to visualize the distribution of genetic biomarkers (12, 53, 54). Endophenotype is an epidemiological term which is used to connect behavioral symptoms with structural phenotypes associated with known genetic causes. It is more robust and stable in the disease process than the broad clinical phenotype. In the field of substance-related disorders, research materials of endophenotype associated to alcohol dependence and addiction have been accumulated. Especially, it has been shown that heavy drinking and alcohol problems contain an endophenotype of low level of response to alcohol (51, 55).

Unlike the term co-occurrence network graph, which depends on the co-occurrence frequency, the burst detection algorithm can identify emergency terms no matter how many times their main article is cited. By applying this method to keyword detection, new research frontiers can be screened before enough references are attracted (26). According to the keywords screened out (Figure 7), burst words related to genetic factors in the field of substance-related disorders include “risk,” “alcohol use disorder,” “genome-wide association,” and “meta-analysis.” Coincidently, these four keywords represent different aspects of research: epidemiology, focus, novel research techniques, and types of academic achievement. Undoubtedly, risk factors have been the most concerned issue by GWAS researchers in recent years. Alcohol use disorder is one of the widespread substance-related disorders, and it is still a research hot spot. With the advent and wide application of GWAS (56), many meta-analyses related to this field have been published in recent years, including some high-quality papers (57–60). It is foreseeable that the research fields represented by the above four keywords will continue to receive academic attention in the future.

## Conclusion

Substance abuse is recognized worldwide as a social problem because it seriously affects the health and social functioning of individuals. The related scientific issues have been the concern of scholars all over the world, and a large amount of human and material resources have been devoted. All original publications from 1997 to 2018 were analyzed by using knowledge mapping tools such as VOSviewer and CiteSpace. We provide objective data on issues such as annual publication maps, international partnerships, research frontiers, citation structure, or co-citation analysis and attempt to reveal their knowledge structure and characteristics.

It is concluded that the US was in a leadership position from the perspective of publication output number, quality of scientific achievement, and global cooperation in the past 22 years in this research field. The advancement of sequencing technology has the potential to revolutionize research methods and appearance. The popularity of GWAS had changed the research methods and improved research efficiency. High-resolution chromosome ideograms of substance-related disorders have been provided by GWAS which bring us a wider vision on genetic factors. Endophenotype is another valuable focus that has gained increasing attention in recent years. It serves as a bridge between genotype and genetic susceptibility. Alcohol use disorder is one of the main branches of research in this field. It is expected that with the help of GWAS, research hot spots will continue to focus on SNPs and related areas in the short term. Although considerable progress has been made in this field in recent years, the etiology and pathogenesis of substance-related disorders are still unclear. We hope this research can provide reference and guidance for colleagues engaged in this field.

## Data Availability Statement

Publicly available datasets were analyzed in this study. This data can be found here: https://apps.webofknowledge.com/.

## Author Contributions

ML and JHu conceived the work. HC collected and downloaded the data. JHe, WD, and NZ helped check and verify data as three independent investigators. YY and JHu performed the visualization work. KW and YD wrote the manuscript. WD helped revise the manuscript and proposed constructive opinions. All authors reviewed and approved the manuscript and have read and agreed to the published version of the manuscript.

## Conflict of Interest

The authors declare that the research was conducted in the absence of any commercial or financial relationships that could be construed as a potential conflict of interest.
