# Supplementary material for: Bibliometric Insights in Genetic Factors of Substance-Related Disorders: Intellectual Developments, Turning Points, and Emerging Trends
Source: Front Psychiatry. 2021 May 31;12:620489. doi: 10.3389/fpsyt.2021.620489 (PMC8200466; doi:10.3389/fpsyt.2021.620489)
Supplement: Supplementary file 1 [file Table_1.DOCX]

| Table S1 Query details |  |  |  |
| --- | --- | --- | --- |
| Substance-Related Disorders | OR | AND | Genetics |
| Drug Abuse | OR |  |  |
| Abuse, Drug | OR |  |  |
| Drug Dependence | OR |  |  |
| Dependence, Drug | OR |  |  |
| Drug Addiction | OR |  |  |
| Addiction, Drug | OR |  |  |
| Substance Use Disorders | OR |  |  |
| Disorder, Substance Use | OR |  |  |
| Substance Use Disorder | OR |  |  |
| Drug Use Disorders | OR |  |  |
| Disorder, Drug Use | OR |  |  |
| Drug Use Disorder | OR |  |  |
| Organic Mental Disorders, Substance-Induced | OR |  |  |
| Substance Abuse | OR |  |  |
| Abuse, Substance | OR |  |  |
| Abuses, Substance | OR |  |  |
| Substance Abuses | OR |  |  |
| Substance Dependence | OR |  |  |
| Dependence, Substance | OR |  |  |
| Substance Addiction | OR |  |  |
| Addiction, Substance | OR |  |  |
| Prescription Drug Abuse | OR |  |  |
| Abuse, Prescription Drug | OR |  |  |
| Drug Abuse, Prescription | OR |  |  |
| Drug Habituation | OR |  |  |
| Habituation, Drug |  |  |  |
